# Impact of Socioeconomic Status on the Diagnosis of Primary Open-Angle Glaucoma and Primary Angle Closure Glaucoma: A Nationwide Population-Based Study in Taiwan

**DOI:** 10.1371/journal.pone.0149698

**Published:** 2016-02-23

**Authors:** Yu-Chieh Ko, De-Kuang Hwang, Wei-Ta Chen, Ching-Chih Lee, Catherine J. Liu

**Affiliations:** 1 Department of Ophthalmology, Taipei Veterans General Hospital, Taipei, Taiwan; 2 Faculty of Medicine, National Yang-Ming University School of Medicine, Taipei, Taiwan; 3 Institute of Clinical Medicine, National Yang-Ming University School of Medicine, Taipei, Taiwan; 4 Department of Ophthalmology, Taichung Veterans General Hospital, Taichung, Taiwan; 5 Neurological Institute, Taipei Veterans General Hospital, Taipei, Taiwan; 6 Department of Otolaryngology, Dalin Tzu-Chi Hospital, Buddhist Tzu Chi Medical Foundation, Chiayi, Taiwan; 7 School of Medicine, Tzu Chi University, Hualian, Taiwan; Duke University, UNITED STATES

## Abstract

**Purpose:**

To understand the impact of socioeconomic status (SES) on the diagnosis of primary open-angle glaucoma (POAG) and primary angle closure glaucoma (PACG) in Taiwan.

**Methods:**

Subjects with glaucoma were identified from the National Health Insurance Research Database of year 2006, which included one million randomly selected insurants. Individuals who had ≥4 ambulatory visits within one year which had the diagnosis code of POAG (ICD-9-CM 365.11 or 365.12) or PACG (365.23) and concurrent prescription of anti-glaucoma medication or surgery were selected. Individual SES was represented by monthly income calculated from the insurance premium. Neighborhood SES was defined based on neighborhood household income averages. Urbanization level of habitation was categorized into 3 levels. The odds ratio of having POAG or PACG in subjects with different SES was evaluated by using multiple logistic regression analysis.

**Results:**

In total, 752 and 561 subjects with POAG and PACG, respectively, who were treated on a regular basis, were identified. The diagnosis of glaucoma was affected by age, gender, frequency of healthcare utilization, individual SES, and urbanization level of habitation. With the adjustment of age, gender, healthcare utilization, neighborhood SES and level of urbanization, subjects with lower income were more likely to be diagnosed as PACG, but less likely as POAG.

**Conclusions:**

Subjects with more frequent healthcare utilization were more likely to be diagnosed with glaucoma. Subjects with low SES were more susceptible to PACG, but subjects with high SES were more likely to be diagnosed as POAG. This information is useful for the design and target participant setting in glaucoma education and screening campaign to maximize the efficacy of limited resources in preventing glaucoma blindness.

## Introduction

Glaucoma is the leading cause of irreversible blindness. Early diagnosis with early intervention may prevent glaucoma blindness.[1.^2^] Because of the asymptomatic nature of most types of glaucoma, early diagnosis of glaucoma is largely dependent on regular and complete ocular examination. Socioeconomic status (SES) may affect glaucoma awareness, eye care service utilization, healthcare seeking behavior and therefore the chance to identify asymptomatic glaucoma.[3–5] Several studies have shown that subjects with lower SES had poorer vision than their better-off counterparts;[6] nevertheless, population-based studies addressing the impact of SES on glaucoma diagnosis are limited. Hospital-based studies revealed that low SES is a risk factor for advanced glaucoma at presentation.[7,8] It is unclear whether the advanced disease at diagnosis in subjects with low SES is the result of delayed diagnosis due to reduced accessibility and use of eye care facility or increased susceptibility.

The limited information about the impact of SES on glaucoma is derived from studies mostly focusing on primary open-angle glaucoma (POAG).[7,8] Subjects with primary angle closure glaucoma (PACG) differ from those with POAG in biometric characteristics.[9] SES, which is associated with biometric characteristics, may therefore affect an individual’s susceptibility to POAG and PACG. Subjects with higher educational attainment tend to be more myopic with longer axial length, which protects them from angle closure but increases their susceptibility to POAG.[10–13]

The National Health Insurance Program of Taiwan aims to provide equal access to healthcare for all citizens. Premium and co-payment for health services is waived for low income earners to ensure care for the disadvantaged subjects. Therefore the inequalities in access to healthcare is minimized across SES.[14] The National Health Insurance Research Database (NHIRD) consists of the registration files and claims data of all the enrollees, which provides us an opportunity to understand the impact of SES on the diagnosis of glaucoma while taking the frequency of healthcare utilization, neighborhood status and urbanization level of habitation into consideration in a population-based setting under a nearly barrier-free healthcare system. The information hereby obtained will be valuable in allocating limited resources to maximize the efficacy of targeted education and screening to prevent glaucoma blindness.

The objective of this study was to understand the impact of individual and neighborhood SES on the diagnosis of POAG and PACG using NHIRD in Taiwan.

## Materials and Methods

### Database

This was a nationwide population-based study. Taiwan began the National Health Insurance program in 1995 which covers >98% of the current 23 million residents and contracts with 97% of Taiwanese medical providers. We used the NHIRD of year 2006 and 2007, a data subsets created for research purpose by systematically and randomly selecting one million subjects, who were covered by the insurance program during 2006 and 2007, and contained all their de-identified enrollment files, claims data and the registry for prescription drugs. There were no differences in age, gender, or healthcare costs between the sampled group and all the enrollees.[15] The accuracy of the claims data of the NHIRD was properly monitored and validated,[16,17] and hundreds of studies based on this dataset have been published in peer-reviewed journals, including ophthalmology-related studies and those focused on glaucoma.[18,19]

Ethical approval was obtained from the Institutional Review Board of Taipei Veterans General Hospital. Informed consent was not obtained because all the information was anonymized and deidentified in the database.

### Study population

From the NHIRD of year 2006, 923202 subjects were included. Subjects with glaucoma were defined as individuals who had ≥4 ambulatory visits within one year from the index date with a diagnosis code of POAG (International Classification of Diseases, 9^th^ Revision, Clinical Modification (ICD-9-CM) 365.11 or 365.12) or PACG (365.23) and concurrent prescription of anti-glaucoma medication or a surgery code of 12.64 (trabeculectomy).

### Measurements

Demographic characteristics including age and gender were recorded. Frequency of healthcare utilization was calculated according to the number of outpatient visits of each subject in year 2006, and stratified into three groups (high, moderate and low) of equal subjects in each group. The SES was evaluated according to individual-level, and neighborhood-level parameters, and the urbanization level of habitation.

#### Individual-level SES

This study used the monthly income calculated from the insurance premium as a proxy for individual SES. The individuals were classified into three groups: (1) low SES: lower than US$571 per month (New Taiwan Dollar (NT$) 20000); (2) moderate SES: between US$571–1141 per month (NT$20000–40000); and (3) high SES: US$1142 per month (NT$40001) or more. The classification was based on the minimum monthly wage for year 2006 in Taiwan (NT$ 15840).

#### Neighborhood-level SES

Neighborhood SES is a contextual factor based on neighborhood household income averages and percentages reported in Taiwan’s Census. In that census, neighborhood household income was measured by township using per capita income, which could be determined based on tax statistics released by Taiwan’s Ministry of Finance. Advantaged and disadvantaged neighborhoods were distinguished based on the median values for neighborhood characteristics, with advantaged neighborhoods having higher-than-median neighborhood household incomes.

#### Urbanization level

The urbanization level of habitation were classified into 7 levels based on 5 indices in Taiwan: population density, percentage of residents with college level or higher education, percentage of residents > 65 years old, percentage of residents who are agriculture workers, and the number of physicians per 100000 people.[20] We recorded the urbanization level of habitation as urban (urbanization level 1), sub-urban (urbanization levels 2–3), or rural (urbanization levels 4–7).

### Statistical analysis

The SAS statistical package, version 9.2 (SAS Institute, Inc., Cary, NC, USA) was used for data extraction and analysis. The univariate association between the diagnosis of POAG or PACG and sociodemographic characteristics, age and gender was analyzed using Chi-square test. The adjusted odds ratio of being diagnosed as POAG or PACG in subjects with different SES was estimated using multiple logistic regression models including age, gender and other sociodemographic factors. A two-sided *p*-value (*p* < 0.05) was used to determine statistical significance.

## Results

The basic characteristics of the sample population and subjects with glaucoma, 752 and 561 subjects with POAG and PACG, respectively, are listed in Table 1. The diagnosis of glaucoma was associated with increased age, and increased frequency of healthcare utilization. Subjects living in urban areas and those with higher SES were more likely to be diagnosed as POAG. PACG was more common in subjects with lower SES. Men were more likely to be diagnosed as POAG than women. (Tables 2 and 3) To evaluate the interaction between individual SES and other sociodemographic factors, the association between neighborhood status, healthcare utilization and urbanization level and the diagnosis of POAG or PACG was analyzed with the stratification of individual SES. The trend of association of these variables with the diagnosis of POAG and PACG did not differ in subjects with different individual SES. (Tables A and B in S1 File) Therefore, the sociodemographic factors were entered simultaneously in the multiple logistic regression model. After adjusting for age, gender, healthcare utilization, neighborhood SES and level of urbanization, subjects with lower SES were more likely to be diagnosed as PACG, and subjects with higher SES were more likely to be diagnosed as POAG. (Tables 2 and 3)

**Table 1:** Basic Characteristics of the Sample Population with Glaucoma (*n* = 1313).

Age (years)	POAG						PACG					
	Male		Female		ALL		Male		Female		ALL	
	Case	Prevalence^a^	Case	Prevalence^a^	Case	Prevalence^a^	Case	Prevalence^a^	Case	Prevalence^a^	Case	Prevalence^a^
**≤39**	79	3.01	52	1.88	131	2.43	5	0.19	3	0.11	8	0.15
**40–49**	61	8.48	53	7.00	114	7.72	12	1.67	13	1.72	25	1.69
**50–59**	61	11.45	65	11.47	126	11.46	34	6.38	49	8.64	83	7.55
**60–69**	69	23.36	82	24.87	151	24.16	66	22.35	110	33.37	176	28.16
**≥70**	141	43.99	89	28.25	230	36.19	120	37.44	149	47.30	269	42.33
**Total**	411	9.15	341	7.19	752	8.15	237	5.28	324	6.83	561	6.08

POAG = primary open-angle glaucoma, PACG = primary angle closure glaucoma.

^a^ 1/10000

**Table 2:** Association between the Diagnosis of Primary Open-Angle Glaucoma and Socioeconomic Status (SES).

Variables	Case (number)	Population (number)	Prevalence (1/10000)	Odds ratio	Adjusted odds ratio^b^	95% CI
**SES**						
Low	297	438,336	6.78	1	1 [reference]	
Moderate	275	330,370	8.32	1.23^a^	1.04	(0.87–1.24)
High	180	154,496	11.65	1.72^a^	1.59 ^a^	(1.29–1.97)
**Age (years)**						
≤39	131	539,512	2.43	1	1 [reference]	
40–49	114	147,684	7.72	3.18^a^	2.72^a^	(2.09–3.54)
50–59	126	109,951	11.46	4.72^a^	3.45^a^	(2.67–4.47)
60–69	151	62,503	24.16	9.94^a^	6.75^a^	(5.30–8.61)
≥70	230	63,552	36.19	14.89^a^	10.72^a^	(8.58–13.38)
**Gender**						
Female	341	474,039	7.19	1	1 [reference]	
Male	411	449,163	9.15	1.27^a^	1.43^a^	(1.23–1.66)
**Neighborhood status**						
Advantaged	417	463,023	9.01	1	1 [reference]	
Disadvantaged	335	460,179	7.28	0.81^a^	0.88	(0.74–1.04)
**Healthcare utilization**						
High	633	328,986	19.24	1	1 [reference]	
Moderate	106	327,028	3.24	0.17^a^	0.04^a^	(0.02–0.06)
Low	13	267,188	0.49	0.03^a^	0.23^a^	(0.19–0.28)
**Urbanization Level**						
Urban	316	301,294	10.49	1	1 [reference]	
Sub-urban	301	417,875	7.20	0.69^a^	0.68^a^	(0.57–0.80)
Rural	135	204,033	6.62	0.63^a^	0.51^a^	(0.40–0.65)

CI = confidence interval

^a^ *p*<0.05

^b^ Adjusted odds ratio was estimated using a multiple logistic regression model with all variables (including personal SES, age, gender, neighborhood status, healthcare utilization, and urbanization level) being included simultaneously.

**Table 3:** Association between the Diagnosis of Primary Angle Closure Glaucoma and Socioeconomic Status (SES).

Variables	Case (number)	Population (number)	Prevalence (1/10000)	Odds ratio	Adjusted odds ratio^b^	95% CI
**SES**						
Low	288	438,336	6.57	1	1 [reference]	
Moderate	213	330,370	6.45	0.98^a^	0.77^a^	(0.64–0.94)
High	60	154,496	3.88	0.59^a^	0.69^a^	(0.51–0.94)
**Age (years)**						
≤39	8	539,512	0.15	1	1 [reference]	
40–49	25	147,684	1.69	11.27^a^	12.50^a^	(5.60–27.86)
50–59	83	109,951	7.55	50.33^a^	48.19^a^	(23.18–100.18)
60–69	176	62,503	28.16	187.73^a^	148.59^a^	(72.90–302.85)
≥70	269	63,552	42.33	282.20^a^	204.12^a^	(100.73–413.64)
**Gender**						
Female	324	474,039	6.83	1	1 [reference]	
Male	237	449,163	5.28	0.77^a^	0.91	(0.77–1.09)
**Neighborhood status**						
Advantaged	283	463,023	6.11	1	1 [reference]	
Disadvantaged	278	460,179	6.04	0.99	0.86	(0.71–1.05)
**Healthcare utilization**						
High	503	328,986	15.29	1	1 [reference]	
Moderate	51	327,028	1.56	0.10^a^	0.05^a^	(0.02–0.10)
Low	7	267,188	0.26	0.02^a^	0.23^a^	(0.17–0.30)
**Urbanization Level**						
Urban	187	301,294	6.21	1	1 [reference]	
Sub-urban	247	417,875	5.91	0.95	0.90	(0.74–1.10)
Rural	127	204,033	6.22	1.00	0.70^a^	(0.53–0.92)

CI = confidence interval

^a^ *p*<0.05

^b^ Adjusted odds ratio was estimated using a multiple logistic regression model with all variables (including personal SES, age, gender, neighborhood status, healthcare utilization, and urbanization level) being included simultaneously.

## Discussion

This study found that subjects who lived in urban area or had more healthcare utilization were more likely to be diagnosed with glaucoma. SES affected the diagnosis of POAG and PACG in different ways; subjects with lower SES were more likely to be diagnosed as PACG, while those with higher SES were more likely to be diagnosed as POAG.

This is the first study to evaluate the impact of SES on the diagnosis of POAG and PACG using nationwide healthcare database. This database enabled us to identify large number of glaucoma patients from various levels of SES, which might be difficult to achieve in population–based screening studies. Since the diagnosis of glaucoma in an administrative database is deemed to be affected by healthcare accessibility and healthcare seeking behavior, the frequency of healthcare utilization was counted and adjusted in this study while estimating the impact of SES on glaucoma susceptibility.

Low SES is a barrier to healthcare access. Previous studies have revealed that subjects with lower SES tend to have less eye care visits, less glaucoma awareness and late presentation of glaucoma.[3–8] Therefore, our finding of a higher proportion of PACG in subjects with lower SES suggests an increased susceptibility to PACG in subjects with lower SES, but not an increased diagnostic rate of PACG. Possible reasons for this association are two-fold. The first, subjects with lower SES may have lower educational attainment, which in turn is associated with less myopic shift of the eye.[10,11] The second, subjects with lower SES may be shorter in height due to maternal and childhood undernutrition.[21] Both hyperopia and short in height are well known risk factors of PACG.[9,22,23] In line with our finding, the Beijing eye study and a population-based study in Mongolia revealed that lower educational attainment is associated with PACG.[12,24] Considering the exponential increase of PACG subjects with aging in our population and that PACG may be the leading cause of glaucoma blindness,[25,26] the elderly and the socioeconomic deprived population should be the target for screening to prevent glaucoma blindness.

The finding that subjects with higher SES are more likely to be diagnosed as having POAG could be explained by either increased diagnostic rates or increased susceptibility to POAG in subjects with higher SES. Eye care visit with comprehensive examination is the only way to identify POAG at an asymptomatic stage. Lack of regular eye care visits is the major risk factor for undiagnosed POAG in several population-based studies.[27,28] Subjects with higher SES may have more eye care visits because of regular preventive eye care visits or increased prevalence of myopia and associated retinopathy necessitating glasses prescription and fundus examination.[10,11,29] Glaucoma may be diagnosed accidentally during these eye care visits. Although frequency of healthcare utilization was adjusted in our model, the specialty and reasons for healthcare visits were unknown. Therefore, the possibility that there is an increased diagnostic rate of POAG in subjects with higher SES due to active eye screening or myopia related disorders cannot be ruled out.

On the other hand, subjects with higher SES may be more susceptible to POAG because of increased prevalence of myopia, a recognized risk factor of POAG.[13] Myopic axial elongation makes the eye more vulnerable to glaucomatous damage because of reduced retinal nerve fiber layer thickness and sclera support at the optic nerve head.[30,31] Although we are not able to sort out the exact cause responsible for increased POAG in subjects with higher SES, emphasizing the association between myopia and glaucoma in public health education may be an effective way to arouse glaucoma awareness for subjects with high SES. It is interesting to note that a hospital-based study in India also found that subjects with higher SES tended to have POAG but not PACG.[32]

Subjects with more healthcare utilization were more likely to be diagnosed with glaucoma. Increased frequency of healthcare utilization may be the result of multiple morbidities or a proactive healthcare seeking behavior, which could not be discriminated in our database. A study investigating healthcare utilization by the elderly in Taiwan found that economic status of the subjects did not affect the rate and costs of ambulatory and in-patient care, while the elderly with higher education used more ambulatory care service, but less in-patient care service.[33] This finding indicates that economic factor may not be a critical barrier to healthcare access in Taiwan, and highlights the importance of education for disease awareness and early intervention to improve treatment outcome. An Australian study also found that the lack of awareness of glaucoma was a major risk for late presentation, rather than the lack of access to care.[34] Several studies have shown that glaucoma awareness, follow-up and medication adherence are affected by education.[3,35] Therefore, in a universal healthcare system with minimal economic barrier to healthcare access, the best way to alleviate socioeconomic burden of glaucoma blindness is to motivate subjects at risk to seek for screening visits. This could be achieved by promoting public education about the risk factors, nature and management of glaucoma.

Subjects living in urban areas are more likely to be diagnosed as having POAG and PACG in this study. Population-based studies found increased prevalence of POAG in urban areas, which may be related to the higher prevalence of myopia in urban areas.[36] On the other hand, easier and more access to healthcare in urban areas may contribute to an increased diagnostic rate of glaucoma in urban than rural areas.

There are several limitations in this study. First, subjects with glaucoma were identified from the database through ICD-9-CM codes reported by ophthalmologists but the diagnostic criteria for glaucoma was not specified in this health care system. The severity of glaucomatous damage was unknown because the data set did not contain related clinical parameters, such as visual field deficits. Therefore, we were unable to evaluate the relationship between SES and disease severity. Second, some subjects with POAG or PACG might have been excluded from this study for the following reasons: non-specific coding of glaucoma by physician with an ICD-9-CM code of 365.9, no concurrent glaucoma medication or intervention, or lack of regular follow-up. We purposely enrolled only subjects with definite diagnosis and regular treatment to avoid enrolling subjects who had the diagnostic code for insurance claim for screening examinations but not for the management of the disease. Third, lens status was not included in this analysis because the ICD-9-CM code of lens status was not detailed in each subjects and the laterality of the diseased eye was unknown. The lack of this information does not affect our results regarding the association between SES and glaucoma, but constrains us from estimating the role of lens extraction on the diagnosis of PACG in this population with prevalent angle closure disease.[25] Fourth, the majority of the insurants were of Chinese race/ethnicity. Last, the reasons for healthcare visits were not included in the database. Therefore, we were not sure whether subjects with higher SES were more likely to have preventive health care in our database.

SES affects the diagnosis of POAG and PACG differently; subjects with lower SES are more likely to be diagnosed as PACG, while those with higher SES were more likely to be diagnosed as POAG. Increased healthcare utilization is associated with the diagnosis of glaucoma. Raising awareness of glaucoma and education for the importance of comprehensive eye examination among people with risk factors, their families and health professionals may improve the rate of glaucoma diagnosis in a universal healthcare system. Socioeconomic deprived subjects and the elderly with limited access to health care may need targeted screening and intervention to prevent glaucoma blindness. These findings should be applied with caution in other populations because of the high prevalence of PACG in Chinese race/ethnicity, and the lack of information about lens status, which may affect an individual’s susceptibility to angle closure.

## Supporting Information

S1 File Association of Sociodemographic Factors and the Diagnosis of Primary Open-Angle Glaucoma (Table A), and Primary Angle Closure Glaucoma (Table B), Stratified by Individual Socioeconomic Status (SES). (DOCX)
