# Supplementary material for: Impact of Socioeconomic Status on the Diagnosis of Primary Open-Angle Glaucoma and Primary Angle Closure Glaucoma: A Nationwide Population-Based Study in Taiwan
Source: PLoS One. 2016 Feb 23;11(2):e0149698. doi: 10.1371/journal.pone.0149698 (PMC4764499; doi:10.1371/journal.pone.0149698)
Supplement: S1 File — (DOCX) [file pone.0149698.s001.docx]

**Table A. Association of Sociodemographic Factors and the Diagnosis of Primary Open-Angle Glaucoma, Stratified by Individual Socioeconomic Status (SES)**

|  | **Low SES** | | | | **Moderate SES** | | | | **High SES** | | | | **Difference in trend (*p* value)** |
| --- | --- | --- | --- | --- | --- | --- | --- | --- | --- | --- | --- | --- | --- |
|  | **Case** | **Population** | **Prevalence** | **Odds ratio** | **Case** | **Population** | **Prevalence** | **Odds ratio** | **Case** | **Population** | **Prevalence** | **Odds ratio** |  |
| **Age(years)** |  |  |  |  |  |  |  |  |  |  |  |  | 0.04 |
| ≦39 | 55 | 330260 | 1.67 | 0.04 | 42 | 144103 | 2.91 | 0.11 | 34 | 65149 | 5.22 | 0.10 |  |
| 40-49 | 16 | 29043 | 5.51 | 0.13 | 49 | 74025 | 6.62 | 0.25 | 49 | 44616 | 10.98 | 0.22 |  |
| 50-59 | 20 | 20662 | 9.68 | 0.23 | 59 | 57430 | 10.27 | 0.38 | 47 | 31859 | 14.75 | 0.29 |  |
| 60-69 | 52 | 21425 | 24.27 | 0.58 | 59 | 30196 | 19.54 | 0.73 | 40 | 10882 | 36.76 | 0.73 |  |
| ≧70 | 154 | 36946 | 41.68 | 1^a^ | 66 | 24616 | 26.81 | 1^a^ | 10 | 1990 | 50.25 | 1^a^ |  |
| **Gender** |  |  |  |  |  |  |  |  |  |  |  |  | 0.27 |
| Female | 151 | 237227 | 6.37 | 1^a^ | 142 | 181682 | 7.82 | 1^a^ | 48 | 55130 | 8.71 | 1^a^ |  |
| Male | 146 | 201109 | 7.26 | 1.14 | 133 | 148688 | 8.94 | 1.14 | 132 | 99366 | 13.28 | 1.52 |  |
| **Neighborhood status** |  |  |  |  |  |  |  |  |  |  |  |  | 0.26 |
| Advantaged | 173 | 208539 | 8.30 | 1^a^ | 130 | 156451 | 8.31 | 1^a^ | 114 | 98033 | 11.63 | 1^a^ |  |
| Disadvantaged | 124 | 229797 | 5.40 | 0.65 | 145 | 173919 | 8.34 | 1.00 | 66 | 56463 | 11.69 | 1.01 |  |
| **Healthcare utilization** |  |  |  |  |  |  |  |  |  |  |  |  | 0.95 |
| High | 254 | 157550 | 16.12 | 1^a^ | 235 | 123478 | 19.03 | 1^a^ | 144 | 47958 | 30.03 | 1^a^ |  |
| Moderate | 38 | 157360 | 2.41 | 0.15 | 34 | 112830 | 3.01 | 0.16 | 34 | 56838 | 5.98 | 0.20 |  |
| Low | 5 | 123426 | 0.41 | 0.03 | 6 | 94062 | 0.64 | 0.03 | 2 | 49700 | 0.40 | 0.01 |  |
| **Urbanization level** |  |  |  |  |  |  |  |  |  |  |  |  | 0.50 |
| Urban | 131 | 131011 | 10.00 | 1^a^ | 93 | 102713 | 9.05 | 1^a^ | 92 | 67570 | 13.62 | 1^a^ |  |
| Sub-urban | 129 | 207752 | 6.21 | 0.62 | 100 | 143641 | 6.96 | 0.77 | 72 | 66482 | 10.83 | 0.80 |  |
| Rural | 37 | 99573 | 3.72 | 0.37 | 82 | 84016 | 9.76 | 1.08 | 16 | 20444 | 7.83 | 0.57 |  |
| **Total** | 297 | 438336 | 6.78 |  | 275 | 330370 | 8.32 |  | 180 | 154496 | 11.65 |  |  |

Prevalence was calculated as 1/10,000

^a^ Reference group

**Table B. Association of Sociodemographic Factors and the Diagnosis of Primary Angle Closure Glaucoma, Stratified by Individual Socioeconomic Status (SES)**

|  | **Low SES** | | | | **Moderate SES** | | | | **High SES** | | | | **Difference in trend (*p* value**) |
| --- | --- | --- | --- | --- | --- | --- | --- | --- | --- | --- | --- | --- | --- |
|  | **Case** | **Population** | **Prevalence** | **Odds ratio** | **Case** | **Population** | **Prevalence** | **Odds ratio** | **Case** | **Population** | **Prevalence** | **Odds ratio** |  |
| **Age (years)** |  |  |  |  |  |  |  |  |  |  |  |  | 0.30 |
| ≦39 | 7 | 330260 | 0.21 | 0.004 | 0 | 144103 | 0 | 0 | 1 | 65149 | 0.15 | 0.01 |  |
| 40-49 | 7 | 29043 | 2.41 | 0.05 | 10 | 74025 | 1.35 | 0.04 | 8 | 44616 | 1.79 | 0.09 |  |
| 50-59 | 17 | 20662 | 8.23 | 0.17 | 46 | 57430 | 8.01 | 0.23 | 20 | 31859 | 6.28 | 0.31 |  |
| 60-69 | 79 | 21425 | 36.87 | 0.77 | 70 | 30196 | 23.18 | 0.66 | 27 | 10882 | 24.81 | 1.23 |  |
| ≧70 | 178 | 36946 | 48.18 | 1^a^ | 87 | 24616 | 35.34 | 1^a^ | 4 | 1990 | 20.10 | 1^a^ |  |
| **Gender** |  |  |  |  |  |  |  |  |  |  |  |  | 0.22 |
| Female | 180 | 237227 | 7.59 | 1^a^ | 127 | 181682 | 6.99 | 1^a^ | 17 | 55130 | 3.08 | 1^a^ |  |
| Male | 108 | 201109 | 5.37 | 0.71 | 86 | 148688 | 5.78 | 0.83 | 43 | 99366 | 4.33 | 1.41 |  |
| **Neighborhood status** |  |  |  |  |  |  |  |  |  |  |  |  | 0.20 |
| Advantaged | 169 | 208539 | 8.10 | 1^a^ | 84 | 156451 | 5.37 | 1^a^ | 30 | 98033 | 3.06 | 1^a^ |  |
| Disadvantaged | 119 | 229797 | 5.18 | 0.64 | 129 | 173919 | 7.42 | 1.38 | 30 | 56463 | 5.31 | 1.74 |  |
| **Healthcare utilization** |  |  |  |  |  |  |  |  |  |  |  |  | 0.92 |
| High | 264 | 157550 | 16.76 | 1^a^ | 188 | 123478 | 15.23 | 1^a^ | 51 | 47958 | 10.63 | 1^a^ |  |
| Moderate | 23 | 157360 | 1.46 | 0.09 | 20 | 112830 | 1.77 | 0.12 | 8 | 56838 | 1.41 | 0.13 |  |
| Low | 1 | 123426 | 0.08 | 0.004 | 5 | 94062 | 0.53 | 0.03 | 1 | 49700 | 0.20 | 0.02 |  |
| **Urbanization level** |  |  |  |  |  |  |  |  |  |  |  |  | 0.41 |
| Urban | 108 | 131011 | 8.24 | 1^a^ | 48 | 102713 | 4.67 | 1^a^ | 31 | 67570 | 4.59 | 1^a^ |  |
| Sub-urban | 148 | 207752 | 7.12 | 0.86 | 80 | 143641 | 5.57 | 1.19 | 19 | 66482 | 5.86 | 1.28 |  |
| Rural | 32 | 99573 | 3.21 | 0.39 | 85 | 84016 | 10.12 | 2.17 | 10 | 20444 | 4.89 | 1.07 |  |
| **Total** | 288 | 438336 | 6.57 |  | 213 | 330370 | 6.45 |  | 60 | 154496 | 3.88 |  |  |

Prevalence was calculated as 1/10,000

^a^ Reference group
